# Transcriptomic Profiling Reveals Biphasic Regulatory Instability and Late-Stage Proteostatic Decline in Aging Mouse Oocytese

**DOI:** 10.3390/genes17010047

**Published:** 2025-12-31

**Authors:** Phuong Thanh N. Dinh, Seung Hwan Lee, Inchul Choi

**Affiliations:** 1 Department of Bio-AI Convergence, Chungnam National University, Daejeon 34134, Republic of Korea; dnphuongthanh1511@gmail.com; 2 Division of Animal and Dairy Sciences, College of Agriculture and Life Sciences, Chungnam National University, Daejeon 34134, Republic of Korea

**Keywords:** oocyte aging, transcriptome, biphasic trajectory, DEG, RNA-seq

## Abstract

**Background:** Maternal aging progressively compromises oocyte competence, yet the precise molecular trajectory across the reproductive lifespan remains insufficiently defined. **Methods:** Here, we mapped the transcriptomic landscape of mouse germinal vesicle (GV) oocytes across three distinct reproductive stages: post-pubertal peak fertility (Young, 8 weeks), fertility decline (Middle, 12 months), and reproductive senescence (Old, 18 months). **Results:** Our bioinformatic analyses reveal that oocyte aging follows a biphasic nonlinear trajectory. The transition from Young to Middle age marked the most profound period of transcriptional destabilization, characterized by 1197 DEGs and extensive perturbation of metabolic and signaling networks. To elucidate the regulatory drivers of this early drift, we performed transcription factor binding site (TFBS) analysis, which identified massive regulatory potential involving master regulators such as LHX8, MYC, and GATA4. Interestingly, despite the predicted extensive TF–target interactions, the mRNA expression levels of these TFs remained stable across age groups. This discrepancy suggests that the observed transcriptional dysregulation is likely associated by age-associated epigenetic modifications that alter chromatin accessibility or binding efficiency, rather than TF depletion. In the subsequent transition from Middle to Old age, the landscape shifted from active perturbation to systemic collapse. This late stage was characterized by mitochondrial respiratory dysfunction and severe proteostatic stress. **Conclusions:** Colectively, our findings define oocyte aging as a biphasic transition from compensatory resistance to systemic collapse. We identify midlife as the critical inflection point of regulatory remodeling, followed by terminal network exhaustion in senescence. This framework provides a molecular foundation for therapeutic and rejuvenation strategies aimed at mitigating age-associated infertility.

## 1. Introduction

Female reproductive aging is a continuous and irreversible biological process characterized by the progressive decline of both ovarian reserve and oocyte competence. With increasing trends in delayed childbearing worldwide, age-associated infertility and adverse reproductive outcomes—including aneuploidy, miscarriage, implantation failure, and congenital anomalies—have become pressing clinical and public health concerns [1,2,3,4]. Central to these outcomes is the oocyte, whose developmental competence is established during oogenesis and determines the success of fertilization and early embryogenesis. Although the physiological manifestations of reproductive aging are well recognized, the molecular mechanisms underlying the transition from peak fertility to reproductive senescence remain incompletely defined.

The germinal vesicle (GV) stage represents a pivotal period during which the fully grown oocyte accumulates a highly specialized maternal mRNA reservoir required for meiotic maturation, fertilization, and early embryo development [5]. Unlike metaphase II (MII) oocytes, which are transcriptionally quiescent, GV oocytes actively transcribe, process, and store mRNAs and regulatory factors that shape downstream developmental competence [6]. Existing transcriptomic studies have demonstrated age-associated changes in maternal-effect gene expression, mitochondrial function, RNA processing, proteostasis, and translational regulation in human and mouse oocytes [7]. However, the majority of these analyses rely on binary comparisons between “young” and “old,” limiting the ability to identify the onset, progression, or nonlinear dynamics of molecular aging. As a result, the specific transcriptomic events that define the onset of fertility decline—often occurring well before overt reproductive failure—remain largely unexplored.

To address these knowledge gaps, we employed a mouse model to systematically investigate gene expression dynamics across three biologically distinct reproductive stages: post-pubertal peak fertility (Young; 8 weeks), the onset of fertility decline (Middle-aged; 12 months), and reproductive senescence (Old; 18 months) [8]. This tri-phasic design allows for the detection of early transcriptional drift that precedes phenotypic decline, the identification of midlife as a potentially discrete molecular state, and the resolution of late-stage degenerative changes characteristic of senescence. By focusing specifically on the GV stage, we captured the primary transcriptional landscape before meiotic resumption modifies the maternal mRNA pool.

In the present study, we performed RNA-sequencing (RNA-seq) and integrative analyses—including differential expression profiling, gene trajectory modeling, functional enrichment, transcription factor binding site analysis, and protein–protein interaction network mapping—to define the transcriptomic trajectory of oocyte aging across the reproductive lifespan. The primary objective was to investigate the molecular basis of this impaired maturation competence by comparing the maternal mRNA reservoir of GV oocytes across the three distinct stages. Specifically, we sought to address: (1) what specific transcriptomic defects are responsible for the total loss of meiotic competence in senescent oocytes, and (2) how the stability of the maternal mRNA pool is compromised as oocytes transition from declining fertility to complete reproductive failure.

## 2. Materials and Methods

All animal studies were performed in accordance with the institutional guideline and prior approval from the Institutional Animal Care and Use Committee (IACUC) of the Chungnam National University (license no. CNU-00702). All chemicals were purchased from Sigma–Aldrich (Sigma-Aldrich, St. Louis, MO, USA) unless otherwise stated.

### 2.1. Sample Preparation, RNA Isolation, and RNA Sequencing

Fully grown germinal vesicle (GV)-intact oocytes were harvested from the ovaries of three distinct age groups of C57BL/6N female mice (KOATECH Pyeongtaek, Republic of Korea): Young (8 weeks; *n* = 7), Middle-aged (12 months; *n* = 9), and Old (18 months; *n* = 22). To initiate follicular development, mice were stimulated by a peritoneal injection of 5 IU of pregnant mare’s serum gonadotropin (PMSG) 46 h before collection. Oocytes were immediately collected in M2 medium containing 2.5 μM milrinone to prevent oocytes from undergoing germinal vesicle breakdown (GVBD) and to ensure the maintenance of the GV-intact stage. To remove surrounding structures, oocytes were briefly treated with Tyrode’s acid to dissolve the zona pellucida and hyaluronidase to eliminate cumulus cells. The denuded GV oocytes were lysed in 10 μL extraction buffer, immediately snap-frozen, and stored at −80 °C until processing. Across the three age groups, a total of 122 (Young), 102 (Middle), and 49 (Old) GV oocytes were retrieved and available for this study (Table 1). For the RNA-seq dataset, three independent biological replicates (*n* = 3) were generated for each age group by pooling these available oocytes. To ensure data consistency and high-quality library preparation, GV oocytes were pooled for each replicate as follows: 30 oocytes per replicate for the Young-aged group, 30 oocytes per replicate for the Middle-aged group, and 15 oocytes per replicate for the Old-age group. The number of pooled oocytes was determined based on the minimum requirements for stable transcriptomic profiling, accounting for the significantly lower oocyte yield observed in senescent mice.

Total RNA was isolated using the PicoPure RNA Isolation Kit (Arcturus, Mountain View, CA, USA) according to the manufacturer’s instructions. RNA amplification was performed with the SMART-Seq v4 Ultra Low Input RNA Kit (Takara Bio, Mountain View, CA, USA) to generate full-length cDNA. Sequencing libraries were prepared using the TruSeq Nano DNA High Throughput Library Prep Kit (Illumina, San Diego, CA, USA). Final libraries were subjected to paired-end sequencing on an Illumina NovaSeq platform (2 × 100 bp). Technical quality was ensured by verifying RNA integrity (RIN > 7.0) before library construction and incorporating internal standards during the sequencing run to monitor error rates. All sequenced samples surpassed the Q30 quality threshold, indicating high technical reliability.

To validate the fidelity of the transcriptomic profiling and ensure that the lower input material in the senescent group did not introduce significant amplification bias, a portion of the total RNA from pooled oocytes was reserved for direct cDNA synthesis prior to SMART-Seq v4 amplification. A comparative analysis via qRT-PCR was performed using both direct and amplified cDNA templates for representative DEGs exhibiting diverse expression trajectories (e.g., up-down, down-up, and up-constant). Detailed primer sequences and the PCR protocol are provided in the Supplementary Materials.

### 2.2. RNA-Seq Dataset Processing and Quality Control

RNA-seq data from Mus musculus germinal vesicle (GV)–stage oocytes were analyzed, with samples categorized into three reproductive-age groups: Young, Middle-aged, and Old. For each age group, three independent biological replicates (*n* = 3) were generated, with each replicate consisting of a pooled library of GV oocytes to ensure robust and representative transcriptomic profiling.

All analyses were performed using the Ensembl GRCm39 genome assembly and annotation. The sequencing data have been deposited in the NCBI BioProject number PRJNA1044526 (https://www.ncbi.nlm.nih.gov/bioproject/PRJNA1044526, accessed on 21 December 2025).

The raw sequences from nine biological replicate libraries (three independent libraries per age group) were subjected to quality control using FastQC v0.11.5 (https://www.bioinformatics.babraham.ac.uk/projects/fastqc/, accessed on 21 December 2025). To ensure assay reliability and monitor for potential technical artifacts, No-Template Controls (NTC) were incorporated during the SMART-Seq v4 amplification process. Adapters were excluded by Cutadapt [9], and low-quality bases (phred score < 33) were trimmed out by Trimmomatic v0.36 [10] using sliding window of 4:15 with a threshold of read length ≥ 36 bp. Mus musculus GRCm39 build reference genome from Ensembl database (http://www.ensembl.org/index.html, accessed on 21 December 2025) was used for mapping the sequenced reads to by STAR aligner v2.7.3a [11]. Mapped reads were counted using GenomicAlignments v1.20.1 [12] and Rsamtools v2.2.3 [13] packages in R to obtain numbers of reads per genes as raw gene expression. To reach closer to the biological meaning of read count, function filterByExpr by EdgeR v3.22.3 package [14] in R was used to filter genes with total reads among all samples smaller than 3 (smallest group size), leaving 17,385 genes for the next process. The read counts were then normalized using TMM method before entering DEG detection to ensure comparability across age groups, accounting for the different number of pooled oocytes (15 in the Old group vs. 30 in Young/Middle groups) [15].

### 2.3. Functional Enrichment Analysis and Protein–Protein Interaction Prediction

DEGs of each comparison (M-Y, O-M, and O-Y) were submitted to the Database for Annotation, Visualization and Integrated Discovery (DAVID) web server [16] for gene ontology (GO) analysis and KEGG-based pathway enrichment. Significant GO terms (*p* ≤ 0.1) belonging to 3 GO categories (biological process (BP), molecular function (MF), and cellular component (CC)) were enriched. Similarly, significantly enriched pathways (*p* ≤ 0.1) were identified. Protein-level interaction predictions for DEGs were generated using the STRING database [17] and visualized as a network in Cytoscape v3.9.1 [18].

### 2.4. Transcription Factor Binding Site (TFBS) Analysis

Because genomic DNA sequence is invariant across age groups, TFBS profiling was applied to assess whether altered transcription factor binding potential could account for age-associated shifts in gene expression. This analysis enabled the identification of TF network instability as a mechanistic contributor to transcriptional drift during oocyte aging. Promoter regions extending 2000 bp upstream of the transcription start site for all DEGs were retrieved using biomaRt [19] from the Ensembl GRCm39 genome. TFBS motif scanning was conducted with JASPAR2022 [20] and TFBSTools [21], using all 251 mouse transcription factor position frequency matrices available in JASPAR. Motif searches were performed with searchSeq() under the thresholds E-value ≤ 5 and *p*-value ≤ 0.01. Genes containing two or more TFBS motifs were analyzed for transcriptional network enrichment.

### 2.5. Statistical Analysis

Differentially expressed genes (DEGs) were identified across the three reproductive-age groups: Young (Y), Middle-aged (M), and Old (O), comparing Middle-aged to Young (M-Y), Old to Middle-age (O-M), and Old to Young (O-Y). To account for the overdispersion and gene-specific variance inherent in high-dimensional count data, we utilized a negative binomial generalized linear model (GLM) implemented in edgeR [14]. The model for gene *i* and sample *j* is defined as:

*Y_ij_*~NB (*μ_ij_,ϕ_i_*), log(*μ_ij_*) = log(L*_j_*) + *X_j_ β_i_*

where *Y_ij_* is the raw count, *μ_ij_* the mean, *ϕ* the gene-specific dispersion, L*_j_* the library size offset for sample *j*, *X_j_* the design matrix encoding age group (Y, M, O), and β*_i_* the vector of coefficients representing the effect sizes for gene i. Likelihood ratio tests compared the full model M_1_ including the stage effect and reduced null model (M_0_). For each gene, the null hypothesis (H_0_) states that gene expression levels do not differ among reproductive-age groups, while the alternative hypothesis (H_1_) posits that at least one group shows a statistically significant difference. The LRT statistic was calculated as:

$$LRT=2\times \left(logL({\hat{\beta }}_{1})-logL({\hat{\beta }}_{0})\right)$$

where $logL({\hat{\beta }}_{1})$ and $logL({\hat{\beta }}_{0})$ are the maximized log-likelihoods under $M_{1}$ and $M_{0}$, respectively. The LRT statistic follows a $\chi^{2}$ distribution with degrees of freedom equal to the difference in model parameters. To control for the false discovery rate across multiple testing analytical layers, we employed a stringent dual-threshold approach: genes with a raw *p* ≤ 0.01 and ∣$\log_{2}\left(Fold\;change\right)|>1$ were considered differentially expressed. These combined criteria ensure that identified DEGs represent biologically significant shifts while mitigating stochastic noise. DEGs were further assigned to temporal expression patterns based on their direction of change across the sequence Young to Middle-aged to Old. Each gene was categorized into one of 8 possible expression patterns: up–up, up–down, down–down, down–up, no-change–up, no-change–down, up–no-change, and down–no-change.

## 3. Results

### 3.1. GV Oocyte Collection and Developmental Competence

GV-stage oocytes were collected from three reproductive-age groups of female mice (Table 1). The Young group (8 weeks; post-pubertal peak fertility) and the Middle-aged group (fertility-decline midlife) yielded 122 and 102 GV oocytes, respectively. In contrast, the Old group—corresponding to human females over approximately 56 years of age and well beyond the end of the mouse reproductive lifespan [1]- produced only 49 GV oocytes, suggesting a profound reduction in oocyte reserve and hormonal responsiveness. Notably, GV oocytes retrieved from 18-month-old mice failed to progress to the MII stage during in vitro maturation [1], further confirming the severe functional decline characteristic of reproductive senescence.

### 3.2. Effect of Aging on GV Oocyte Gene Expression

**Figure 1:**
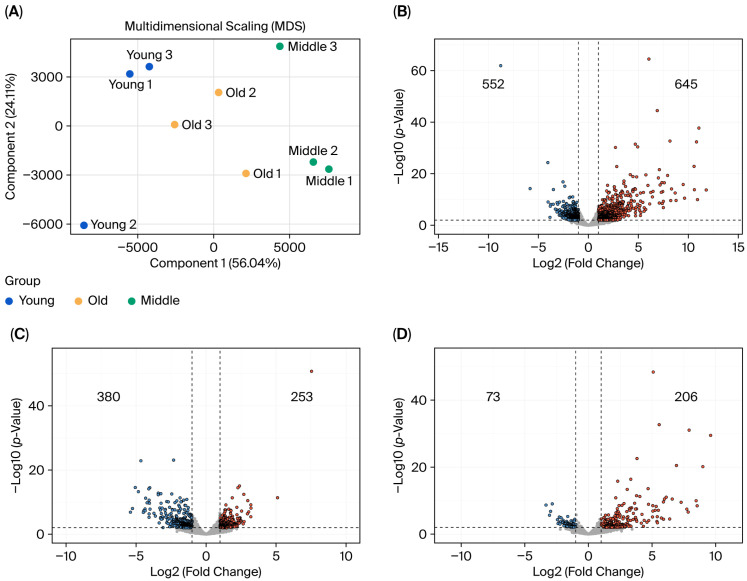
Multidimensional scaling (MDS) and volcano plots of GV-stage oocyte transcriptomes across three reproductive age groups. (**A**) MDS plot depicting global transcriptomic variation among Young (8 weeks; orange), Middle-aged (12 months; green), and Old (18 months; navy) GV-stage mouse oocytes. Volcano plots of age-related differential gene expression in GV oocytes. (**B**) Young vs. Middle, (**C**) Middle vs. Old, and (**D**) Young vs. Old. |log_2_FC| ≥ 1 and *p* ≤ 0.01. Red dots indicate upregulated genes and blue dots indicate downregulated genes.

Multidimensional scaling (MDS) analysis demonstrated a clear separation of GV oocyte transcriptomes according to maternal age. Along the first dimension, Young GV oocytes clustered distinctly on the left, whereas Middle-aged oocytes formed a separate cluster on the right. Interestingly, Old GV oocytes occupied an intermediate position between these two groups rather than following a linear progression further to the right. This spatial structure indicates that while the transcriptomes cluster strictly according to reproductive age, the underlying molecular trajectory is fundamentally nonlinear, highlighting midlife as a major inflection point in the aging process (Figure 1A).

To identify genes associated with maternal aging and impaired oocyte competence, we performed pairwise differential expression analysis across the three reproductive age groups. The Middle vs. Young comparison produced the largest transcriptomic shift, yielding 1197 DEGs, consisting of 552 downregulated and 645 upregulated genes (|log_2_FC| ≥ 1, *p* ≤ 0.01) (Figure 1B). In contrast, substantially fewer DEGs were detected in the Old vs. Middle comparison (633 genes; 380 downregulated, 253 upregulated) and the Old vs. Young comparison (279 genes; 73 downregulated, 206 upregulated) (Figure 1C,D, Table S1). These results indicate that the major remodeling of the GV oocyte transcriptome occurs during the transition from peak fertility (Young) to midlife (Middle), whereas progression from midlife to reproductive senescence involves more selective and limited transcriptional changes.

### 3.3. Temporal Expression Patterns Using DEG

**Figure 2:**
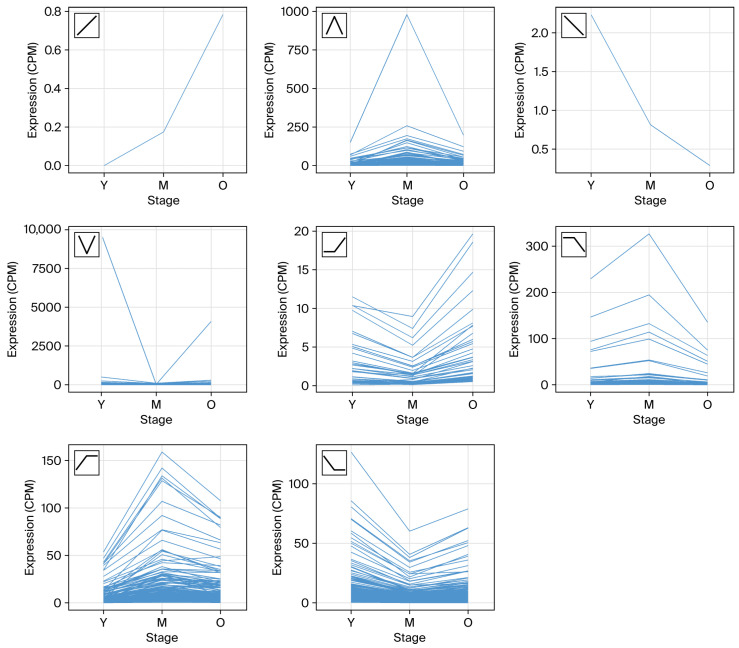
Temporal gene expression patterns across Young, Middle, and Old GV-stage oocytes. Each trajectory line represents normalized expression changes in individual DEGs from Young (starting point) to Middle and then to Old. The specific pattern types (Up–Up, Down–Down, Up–Down, Down–Up, Up–Constant, Down–Constant, Constant–Up, Constant–Down) are shown in the small box at the upper-left corner. These trajectories indicate that GV-oocyte aging follows nonlinear, stage-specific expression dynamics, with midlife representing the major inflection point.

To further characterize how gene expression changes across reproductive aging, all DEGs were classified into eight temporal expression patterns based on their directional changes across the Young, Middle, and Old groups (Figure 2, Table S2). These patterns captured progressive, nonlinear, and stage-specific transcriptional trajectories. Only two genes exhibited strictly monotonic changes across aging: one gene in the Up–Up pattern and one gene in the Down–Down pattern, indicating that continuous activation or repression is extremely rare during oocyte aging. In contrast, the largest gene clusters displayed midlife-onset remodeling. The Up–Constant pattern (371 genes) consisted of transcripts that increased sharply in Middle-aged oocytes and then stabilized in Old oocytes. Similarly, the Down–Constant pattern (317 genes) included genes that showed a marked decrease at midlife followed by no substantial change thereafter. Two nonlinear reversal patterns were also observed. The Up–Down pattern (256 genes) showed a transient increase at midlife followed by downregulation in Old oocytes, whereas the Down–Up pattern (193 genes) reflected midlife suppression with partial recovery in senescence. Finally, age-related changes that emerged only between Middle and Old were captured by the Constant–Up (53 genes) and Constant–Down (114 genes) patterns.

Together, these eight trajectories demonstrate that transcriptomic aging in GV-stage oocytes is highly nonlinear and dominated by midlife-driven reprogramming, rather than progressive, stepwise deterioration. The predominance of the Up–Constant and Down–Constant clusters highlights midlife as the key inflection point where the majority of transcriptional destabilization occurs, consistent with the DEG and functional enrichment analyses.

### 3.4. Functional Enrichment Analysis of DEGs

Following the identification of DEGs and their temporal expression patterns, we performed Gene Ontology (GO) and KEGG pathway enrichment analyses to functionally characterize the transcriptomic shifts driving oocyte aging (Tables S3 and S4). The functional landscape revealed distinct biological features corresponding to the biphasic aging trajectory.

The transition from peak fertility to midlife (Young vs. Middle) was marked by the most extensive functional reorganization, yielding 439 enriched GO terms and 48 enriched KEGG pathways. This phase was characterized by the active regulation of cell survival and homeostatic processes. Specifically, the most significantly enriched biological processes included the “negative regulation of apoptotic process” (e.g., *Birc7*, *Timp1*) and “regulation of cell population proliferation” (e.g., *Ccnd1*, *Cdkn1b*), suggesting a robust effort to balance cell survival against early aging stress. In parallel, key signaling pathways such as the “Phospholipase D signaling pathway” (e.g., *Adcy1*, *Ptk2b*) and metabolic adjustments involving “Taurine and hypotaurine metabolism” were significantly enriched, reflecting a broad physiological remodeling to maintain oocyte competence.

In contrast, the transition to reproductive senescence (Middle vs. Old) involved a more targeted decline, associated with 136 GO terms but only 5 KEGG pathways. A critical finding in this phase was the specific enrichment of “microtubule cytoskeleton organization” (GO:0000226), involving genes such as *Cntn2*, highlighting that the structural instability of the spindle apparatus—a hallmark of aged oocytes—is a late-onset defect. Furthermore, the enrichment of “intracellular signal transduction” (e.g., *Mapkapk2*, *Plek*) suggests a dysregulation in the oocyte’s ability to process environmental cues in the final stages of reproductive life.

Comparing the endpoints of the reproductive lifespan (Young vs. Old) revealed a highly specific molecular signature dominated by protein homeostasis failure. The top enriched terms were exclusively related to “Antigen processing and presentation” (e.g., *H2-Aa*, *H2-Ab1*, *Cd74*) and “MHC class II protein complex”. As oocytes are not immune effectors, this specific enrichment—involving proteasome subunits like *Psmb8* and *Psmb9*—indicates an upregulated ubiquitin-proteasome system in response to the accumulation of damaged or misfolded proteins, confirming a state of severe proteostatic stress in senescent oocytes.

**Figure 3:**
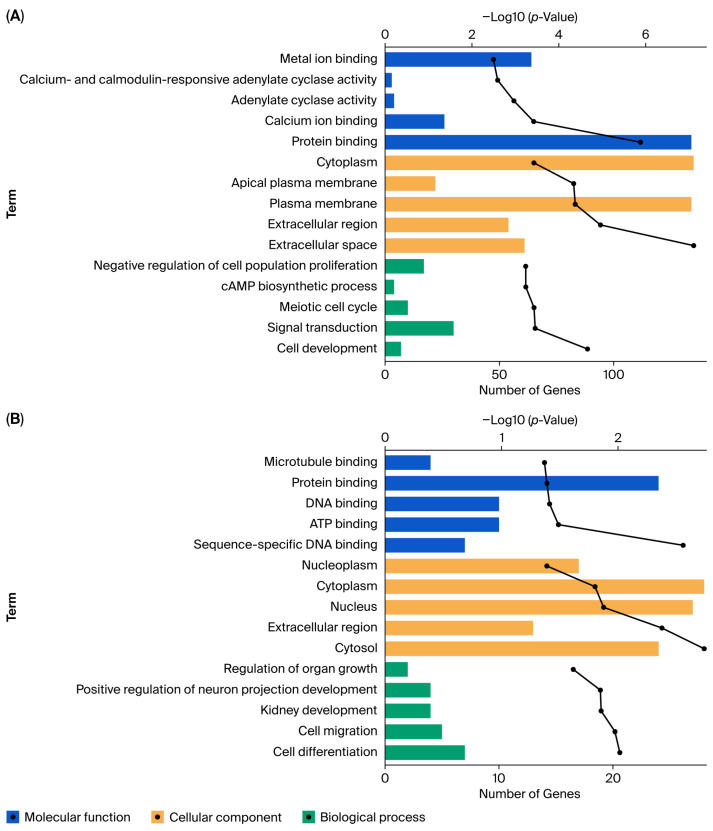
Functional characterization of nonlinear transcriptomic patterns associated with biphasic oocyte aging. (**A**) Top 5 enriched Gene Ontology (GO) terms for the Up/Down-Constant (Early Transition) pattern. This phase is characterized by extensive remodeling of the microenvironment and signaling machinery, with significant enrichment in structural terms and signaling-related functions. (**B**) Top 5 enriched GO terms for the Constant-Up/Down (Late Onset) pattern. This profile reveals a late-stage breakdown in regulatory networks, highlighted by terms related to transcriptional control and developmental instability.

To dissect the nonlinear dynamics of aging, we analyzed the ‘Up/Down-Constant’ and ‘Constant-Up/Down’ expression patterns (Tables S5 and S6). The Up/Down-Constant pattern (Early Transition) represents genes whose expression shifts between Young and Middle age and then plateaus (Figure 3A), reflecting the initial destabilization of oocyte maturation machinery through “cAMP signaling pathway” and “calcium ion binding” (e.g., Adcy1, Adcy5, Cacna1c) (Table S6). In contrast, the Constant-Up/Down pattern (Late Onset) comprises genes whose expression levels remain stable during the transition from Young to Middle age, but undergo a significant transcriptional shift specifically in the Old group. This late-stage profile reveals a breakdown in regulatory networks, particularly in meiotic progression and transcriptional control (e.g., Foxc1, Batf3) (Figure 3B). Furthermore, KEGG analysis for this phase identified a specific enrichment in “Arachidonic acid metabolism” involving Cyp2c family genes (Table S7).

### 3.5. Transcription Factor Binding Site (TFBS) Enrichment Analysis

To determine whether the age-dependent DEGs identified in Young, Middle, and Old GV oocytes are coordinated by transcription factor expression, we performed a transcription factor binding site (TFBS) enrichment analysis on the 2 kb upstream promoter regions of DEGs. This analysis revealed a distinct hierarchy in regulatory complexity across reproductive stages.

The most extensive regulatory remodeling was observed during the transition from peak fertility (Young) to midlife (Middle). The Young vs. Middle comparison exhibited the highest density of regulatory interactions, identifying 10,915 predicted binding events. In contrast, the Middle vs. Young comparison yielded 5356 bindings, while the Young vs. Old comparison showed 2588 bindings (Table S7).

We further analyzed the frequency of specific TFs and identified genes regulated by combinatorial control (genes targeted by >2 TFs). We then detected 16 enriched binding motifs corresponding to 12 unique transcription factors. Specifically, the binding motifs for LHX8 (MA0705.1), GATA factors (GATA1, MA0035.3; GATA4, MA0482.1), TCF3 (MA0522.1), BHLHE40 (MA0464.1), TCF12 (MA0521.1), RXRA (MA0512.1), and MYC (MA0499.1, MA0147.2) were significantly enriched. Remarkably, although these TFs were expressed in GV oocytes, none were identified as DEGs. This indicates that the mRNA abundance of these key upstream regulators remains stable throughout reproductive aging, despite the significant fluctuation in their downstream target genes.

### 3.6. Topological Architecture and Functional Modules of PPI Networks

**Figure 4:**
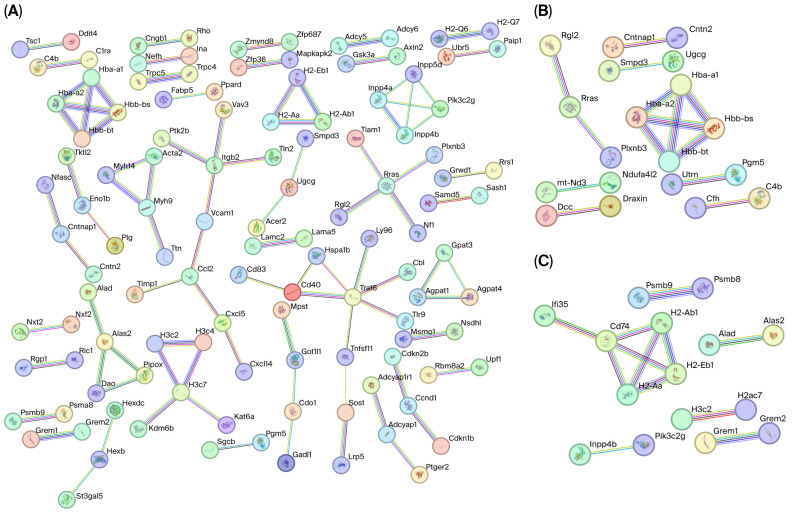
Topological reorganization of protein–protein interaction (PPI) networks. Nodes represent proteins, and edges represent high-confidence functional associations. (**A**) Young vs. Middle: This network displays the highest connectivity. (**B**) Middle vs. Old: A marked reduction in connectivity is observed, indicating selective deterioration of functional modules rather than broad reorganization. (**C**) Young vs. Old: The network is sparse and fragmented.

To map the functional interactome of differentially expressed genes (DEGs), we constructed Protein–Protein Interaction (PPI) networks for each age comparison using the STRING database. Topological analysis revealed a significant stage-specific reduction in network density across the reproductive lifespan (Table S8). The Middle vs. Young (MY) network exhibited the highest complexity, comprising 769 nodes and 90 edges (enrichment *p* = 1.98 × 10^−5^). In contrast, the Middle vs. O (MO) network contained 368 nodes with 14 edges, while the Young vs. Old (YO) network was the sparsest, consisting of only 182 nodes and 12 edges (Figure 4).

Within these networks, we identified specific gene clusters critical for reproductive competence. In the context of mitochondria and metabolism, the YM network was enriched with 49 genes associated with lipid metabolism and autophagy, featuring key regulators such as Acacb, Acadl, and Atg2a. Conversely, the MO and YO networks highlighted core components of the electron transport chain and ATPase machinery, identifying Atp8a2 and the cytochrome c oxidase subunit Cox4i2 as central nodes. Regarding genomic integrity, meiotic regulators were predominantly identified in the YM comparison, which contained a cluster of 27 genes including Dmc1, Kifc1, and Marf1. The MO network specifically highlighted Smc2, a protein involved in the structural maintenance of chromosomes. Furthermore, a distinct signature of cellular stress response and proteostasis was observed. A large cluster of 88 stress-response genes was detected in the YM network (e.g., Casp6, Card14), whereas the OM network retained a reduced cluster of 43 genes, including Ccnb1ip1, Egln3, and the chaperone Clgn.

## 4. Discussion

### 4.1. Reframing the Biphasic Trajectory of Oocyte Aging

A central strength of this study lies in the inclusion of bona fide senescent oocytes derived from aged mice (>18 months), a sample that is both technically and biologically challenging to obtain. While 18-month-old subjects yielded only 49 GV oocytes from 22 mice—a profound reduction in reserve—this stage is essential to resolve the molecular transition from reproductive decline to terminal reproductive exhaustion. Most existing studies rely on binary designs that overlook the nonlinear dynamics of aging. Our data reveal that the Middle group (12 months) represents a sub-fertility transition characterized by a compromised meiotic potential; at this stage, oocytes retain a suboptimal maturation capacity despite cumulative stress [7]. In contrast, the Old group reflects a functional reproductive senescence. The MDS analysis supports this; the spatial positioning of Old oocytes reflects a terminal regulatory collapse and the disappearance of the compensatory networks established at midlife, rather than a return to the peak fertility seen in the Young group.

Our data demonstrate that oocyte aging does not proceed through linear accumulation of transcriptional perturbations. Instead, it follows a biphasic trajectory composed of two biologically distinct phases. The first phase (Young to Middle) is characterized by extensive transcriptional remodeling and network instability, consistent with an adaptive response to aging-associated stress (Phase 1) [1,3]. The second phase (Middle to Old) is marked by the exhaustion of these buffering mechanisms. This biphasic model explains why senescence is not a mere linear extension of midlife changes; instead, it represents a loss of regulatory coherence where the massive compensatory efforts of midlife vanish, leading to the terminal reproductive failure and meiotic arrest observed in 18-month-old oocytes [22].

Our MDS analysis provides a conceptual framework for this distinction. Middle-aged oocytes (12 months) occupy a transcriptomic space consistent with an active resistant state, in which extensive remodeling occurs to compensate for accumulating molecular stress while maintaining meiotic competence. In contrast, senescent oocytes (18 months) do not represent a further extension of this trajectory. Instead, they shift toward a distinct coordinate, reflecting the exhaustion and collapse of compensatory regulatory networks that were actively remodeled at midlife. This model explains why senescent oocytes do not represent an extension of the Middle-stage transcriptome. Rather than amplifying midlife changes, senescence reflects loss of regulatory coherence, producing the nonlinear spatial structure observed in the MDS analysis (Figure 1A). Importantly, this interpretation is concordant with functional outcomes: Middle oocytes retain partial meiotic competence, whereas Old oocytes exhibit near-complete meiotic arrest.

Specific molecular shifts exemplify this network disintegration. For instance, the downregulation of Mtfr2 and loss of Hmgn3 compromise mitochondrial quality control and chromatin integrity, respectively [23,24]. Additionally, the aberrant fluctuation of Ube2s—downregulated at midlife but upregulated in senescence—destabilizes the ubiquitin-proteasome system (UPS). Given that UPS deficiency is a known driver of meiotic arrest and spindle defects, the dysregulation of Ube2s identified in our study offers a reliable molecular explanation for the terminal decline in meiotic competence [25,26].

### 4.2. Temporal Expression Trajectories Reveal Aging Mechanisms Beyond Pairwise DEG Analysis

By classifying genes into eight distinct expression trajectories, we demonstrate that transcriptional dysregulation reflects cumulative temporal dynamics rather than isolated events. This approach resolves mechanisms that conventional pairwise analysis misses, particularly identifying how attenuated meiotic efficiency develops. The Up-Constant and Down-Constant patterns identify genes whose dysregulation emerges during the midlife transition and persists through senescence as permanent molecular defects. A prominent case is Adcy2 (Adenylate Cyclase 2) [27,28,29]. Given that meiotic resumption requires a rapid decrease in intra-oocyte cAMP, the sustained overexpression of Adcy2 established at midlife may create a “cAMP barrier,” mechanistically explaining the high rates of maturation failure in senescent oocytes [30]. Conversely, Constant-Up trajectories identify late-onset failures such as Gpr55 (G Protein-Coupled Receptor 55). Typically downregulated during maturation, the accumulation of Gpr55 specifically in Old oocytes likely disrupts spindle organization and further compromises meiotic progression [31].

In contrast to the active remodeling of midlife, the transition to senescence (Old) was marked by network disintegration and specific functional failures. The enrichment of antigen-processing and MHC class II pathways (e.g., *Cd74*, *Psmb8*) in the Old group likely reflects a “proteostatic collapse,” where the oocyte’s capacity to degrade misfolded proteins is overwhelmed. This interpretation is supported by recent multi-omics evidence linking aging to ribosome pausing and proteostasis impairment [1]. Furthermore, the appearance of electron transport chain components like *Cox4i2* and *Atp8a2* in the late-stage networks signals a failure of the earlier metabolic adaptation, leading to cumulative oxidative damage and bioenergetic deficit [32,33]. This aligns with the well-established “mitochondrial theory of aging” but refines the timeline, pinpointing respiratory dysfunction as a late-stage consequence rather than an early driver.

### 4.3. DEG Regulation by Epigenetic Changes

Our TFBS analysis identified stable mRNA levels for transcription factors like LHX8, MYC, and GATA4, despite significant fluctuations in their target genes. This uncoupling strongly suggests that transcriptional drift is driven by alterations in chromatin accessibility that impair TF binding efficiency rather than TF expression. We here propose that this “epigenetic drift” is mechanistically linked to the age-associated shifts in cellular metabolism. Epigenetic modifying enzymes are strictly dependent on metabolites as cofactors or substrates—for example, histone acetyltransferases require Acetyl-CoA, and sirtuins (histone deacetylases) rely on NAD+ levels [34,35]. In our study, the transition to midlife was characterized by extensive remodeling of metabolic networks, including the upregulation of lipid metabolism regulators like Acacb and Acadl [36,37]. Since Acacb regulates the flux of Acetyl-CoA, its dysregulation at midlife likely disrupts the intracellular pool of Acetyl-CoA available for histone acetylation, leading to aberrant chromatin remodeling.

### 4.4. Candidate Genes by Integrated Transcriptome Analysis

To verify the biological relevance of our transcriptomic profiling, we first examined whether the identified candidate genes have established roles in oocyte biology. Several ovary-predominant genes, including Dync1i1, Plk2, Manba, and Slc39a8, have been previously implicated in oocyte maturation and early developmental competence, supporting the biological validity of our dataset. In particular, Dync1i1 dysregulation is consistent with recent studies demonstrating that DYNC1I1 interacts with CLASP1 to coordinate PLK1-mediated spindle organization and cytokinesis in mouse oocytes [38]. Likewise, Plk2 is a known regulator of centriole duplication and spindle checkpoint signaling, and its altered expression in aged oocytes likely reflects conserved mechanisms of cytoskeletal instability associated with reproductive aging [39]. The identification of Manba, a key enzyme involved in lysosomal glycoprotein degradation, further points to age-associated defects in metabolic clearance and cellular homeostasis [40].

Given that senescent oocytes are obtained in limited numbers, a potential concern was whether the reduced oocyte input in the Old group compared to the Young and Middle groups could compromise sensitivity or introduce amplification-related bias, thereby exaggerating signals of transcriptional collapse. To directly address this issue, we performed independent validation using unamplified direct cDNA synthesis from a reserved portion of pooled RNA prior to SMART-Seq v4 amplification. Comparative qRT-PCR analyses using both direct and amplified cDNA templates demonstrated highly concordant expression patterns across representative DEGs exhibiting diverse temporal trajectories, including up–down, down–up, and up–constant profiles (Supplementary Figure S1). Importantly, both the direction and relative magnitude of expression changes were preserved across templates, indicating that the observed transcriptomic patterns are not artifacts of amplification or reduced input material. These validation steps, together with TMM normalization and the inclusion of No-Template Controls, confirm that the biphasic transcriptional instability observed in senescent oocytes reflects a robust biological phenomenon rather than a technical limitation. Collectively, these results reinforce the reliability of our integrated transcriptomic analysis and support the interpretation that dysregulation of key meiotic, metabolic, and cytoskeletal regulators contributes to the irreversible loss of oocyte maturation competence during reproductive senescence.

Beyond individual candidate genes, integration of TFBS, DEG, and PPI analyses enabled the identification of higher-order regulatory hubs whose dysregulation likely precipitates the loss of reproductive potential. Among these, the meiotic regulator Marf1, identified as a hub gene in the Middle-aged group, plays a critical role in maintaining maternal mRNA stability and oocyte competence [41]. Likewise, altered expression of Kifc1 and Smc2 links early transcriptional drift to defects in spindle organization and chromosomal stability [42,43]. Notably, functional characterization of these genes has largely been confined to non-mammalian systems such as Drosophila, Xenopus, and yeast. Our data demonstrate that these evolutionarily conserved regulators undergo significant transcriptional alteration in the mouse oocyte aging trajectory, providing direct evidence that their roles extend to mammalian oocyte competence. This finding underscores the added value of integrative network-based analysis in uncovering conserved, yet previously underappreciated, regulators of reproductive aging.

### 4.5. Study Limitations

Despite the rigor of our tri-phasic design and technical validations, several inherent limitations must be considered. First, while transcriptomic profiling provides a high-resolution blueprint of molecular aging, mRNA abundance may not perfectly correlate with final protein levels or functional activity due to complex post-transcriptional and translational regulation in oocytes.

Second, the restricted sample size of the senescent group, necessitated by the extreme scarcity of 18-month-old oocytes, presents a potential risk of technical bias, particularly regarding the detection sensitivity of low-abundance transcripts. We acknowledge that this technical constraint could potentially influence the perceived scale of the “systemic collapse” observed in the Old group. However, our comparative validation—demonstrating a high correlation between direct (unamplified) cDNA and RNA-seq templates—strongly suggests that the identified trajectories are a genuine reflection of reproductive aging rather than methodological artifacts.

Finally, while well-known key regulators of oocyte maturation were successfully identified in our analysis, the specific roles of other newly discovered candidate genes in driving meiotic failure remain to be definitively characterized through functional loss-of-function (LOF) or gain-of-function (GOF) experiments. Future research integrating proteomics and translatomics will be essential to establish the precise causal links between these transcriptomic drifts and the observed functional phenotypic decline.

## 5. Conclusions

Collectively, our study establishes a comprehensive molecular framework for understanding oocyte aging as a nonlinear, biphasic process. We delineate an early phase of compensatory resistance during the sub-fertility transition, characterized by extensive transcriptional remodeling and metabolism-coupled epigenetic drift aimed at maintaining meiotic competence. In stark contrast, reproductive senescence represents a systemic collapse of the gene regulatory networks governing proteostasis, mitochondrial function, and meiotic progression, occurring once these regulatory and buffering networks are exhausted.

By identifying the specific regulatory hubs that characterize these functional transitions, we highlight midlife as a critical window of regulatory plasticity. The biological relevance of the identified genes, whose functions align closely with the observed meiotic arrest, further reinforces the fidelity and robustness of our dataset. These findings offer actionable targets for future therapeutic modulation and rejuvenation strategies, potentially providing new avenues for mitigating age-associated infertility and extending the female reproductive lifespan.

## Figures and Tables

**Table 1:** Summary of young, middle, and old GV oocyte collection.

	No. of GV Oocytes	Ages	No. of Mice	Body Weight (g)
Young	122	8 weeks	7	20.37 ± 0.63
Middle	102	12 months	9	31.34 ± 0.23
Old	49	18 months	22	39.68 ± 7.19

## Data Availability

All data generated during this study are published within this article or deposited in NCBI BioProject number PRJNA1044526.

## Supplementary Materials

The following supporting information can be downloaded at: https://www.mdpi.com/article/10.3390/genes17010047/s1, Table S1. mouse_DEG, Table S2. mouse GE pattern, Table S3. GO merged DEG, Table S4. KEGG merged DEG, Table S5. GO merged nonlinear, Table S6. KEGG merged nonlinear, Table S7. mouse_TFBS_list_freq, Table S8. Mouse PPI, Table S9. Sequences of Primers for qRT-PCR, Figure S1. Validation of DEG using qRT-PCR.

## Abbreviations

The following abbreviations are used in this manuscript:

GV	Germinal Vesicle
MII	Metaphase II
RNA-seq	RNA-sequencing
IACUC	Institutional Animal Care and Use Committee
PMSG	Pregnant Mare Serum Gonadotropin
TMM	Trimmed Mean of M-values
DEGs	Differentially Expressed Genes
GLM	Generalized Linear Model
DAVID	Database for Annotation, Visualization and Integrated Discovery
GO	Gene Ontology
KEGG	Kyoto Encyclopedia of Genes and Genomes
BP	Biological Process
MF	Molecular Function
CC	Cellular Component
PPI	Protein–Protein Interaction
TF	Transcription Factor
TFBS	Transcription Factor Binding Site
MDS	Multidimensional Scaling
FC	Fold Change
GVBD	Germinal Vesicle Breakdown
cAMP	Cyclic Adenosine Monophosphate
UPS	Ubiquitin-Proteasome System
MHC	Major Histocompatibility Complex
ATAC-seq	Assay for Transposase-Accessible Chromatin using sequencing
Ribo-seq	Ribosome Profiling sequencing
