# Supplementary material for: Transcriptomic Profiling Reveals Biphasic Regulatory Instability and Late-Stage Proteostatic Decline in Aging Mouse Oocytese
Source: Genes (Basel). 2025 Dec 31;17(1):47. doi: 10.3390/genes17010047 (PMC12841155; doi:10.3390/genes17010047)
Supplement: Supplementary file 1 [file genes-17-00047-s001.zip › genes-4049979-supplementary/DATA FILES/Supplemental data 9.pdf]

## Supplemental Materials and Methods

### qRT-PCR Validation of Transcriptomic Data

To validate the transcriptomic profiles obtained from RNA-seq and to ensure the absence of amplification-induced bias—particularly concerning the reduced oocyte input in the senescent group—quantitative real-time PCR (qRT-PCR) was performed using unamplified (direct) cDNA. Total RNA was isolated from pooled oocytes (30 oocytes each for Young and Middle-aged groups; 15 oocytes for the Old group) using the PicoPure RNA Isolation Kit (Arcturus, Mountain View, CA, USA). Direct cDNA was synthesized from a reserved portion of the total RNA using the QuantiNova cDNA Synthesis Kit (Qiagen, Hilden, Germany).

qRT-PCR was conducted on a StepOne Plus Real-Time PCR System (Applied Biosystems, Foster City, CA, USA) using SFCgreen® (BIOFACT, Daejeon, Republic of Korea). We targeted representative DEGs showcasing diverse expression trajectories, including *Manba*, *Cttnbp2*, *Dync1i1*, *Tceal8*, and *Slc4a3*. The oocyte-specific histone variant ***H1foo*** was employed as an internal reference for normalization. Relative gene expression levels were determined using the $2^{-\Delta\Delta Ct}$ method, with the Young group (8 weeks) serving as the baseline (Relative Quantity, RQ = 1.0).

Supplemented Table 1. Sequences of Primers for qRT-PCR

| Gene Name | Forward (5' to 3') | Reverse (5' to 3') | Product size (bp) | Accession number |
|---|---|---|---|---|
| *H1foo* | CGAAACCGAAAGAGGTCAGAA | CTCCCACTGCGTTTGACCTT | 100 | NM_138311.3 |
| *Manba* | CAACTTCATCCGAAAGGCACAG | GATTCCCTGAGAGGGGAAGGA | 70 | NM_027288.3 |
| *Tceal8* | CAGTTCCCCAGTAGCAGCTC | GCTCAAAAGATCAAGAGTGCTGG | 75 | NM_025703.3 |
| *Dync1i1* | AGCATTGGCATATCACCGGA | TTGTGCAGTCGTCTCCTTGT | 104 | NM_001191027.1 |
| *Cttnbp2* | TCTCTGTCGAAGCAACAGTCC | CCCAAAGAGAGTCTGTGGTCC | 74 | NM_080285.2 |
| *Slc4a3* | CCCTGATGCTAAGGAGAAGCC | TCCTCCGGGATCTTCTCCAG | 90 | NM_001357149.1 |

SF.1.

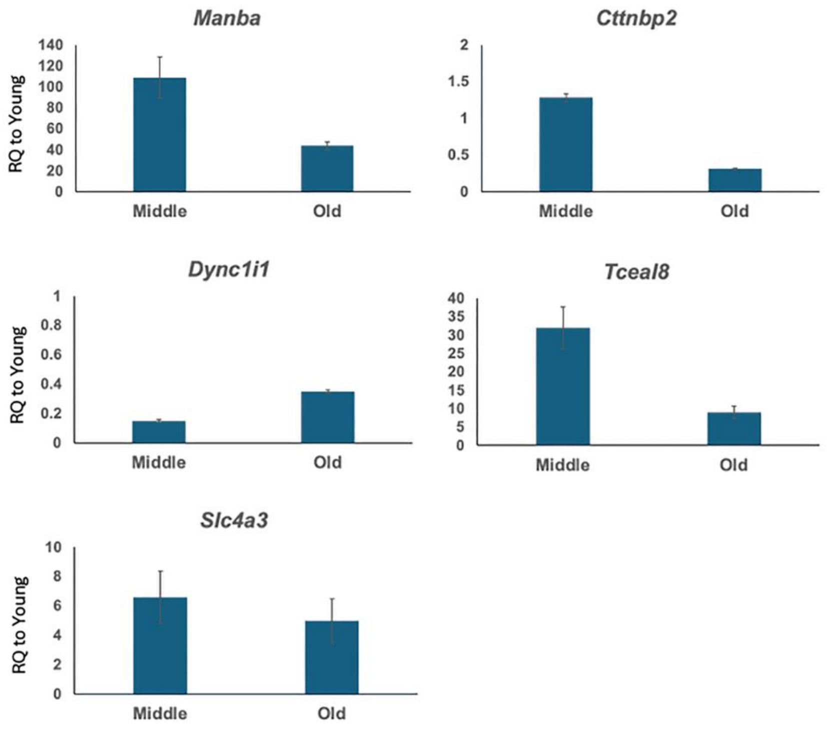


**Supplemental Figure S1. Technical validation of RNA-seq data via qRT-PCR using direct cDNA.** To verify that the reported transcriptomic shifts were not artifacts of the SMART-Seq v4 amplification process or library pooling, relative expression levels of five representative DEGs (*Manba*, *Cttnbp2*, *Dync1i1*, *Tceal8*, and *Slc4a3*) were assessed in Middle-aged (12 months) and Old (18 months) GV oocytes. Expression levels were normalized to the internal oocyte-specific control ***H1foo***. Values are presented as Relative Quantity (RQ) compared to the Young group (normalized to 1.0). Error bars represent the standard error of the mean (SEM) from technical replicates.
